# 14例原发性肺淋巴上皮癌的临床病理分析

**DOI:** 10.3779/j.issn.1009-3419.2024.101.29

**Published:** 2024-11-20

**Authors:** Yixuan FANG, Anzhe WANG, Lumin SHEN, Xiao YUAN, Yu KONG

**Affiliations:** 215006 苏州，苏州大学附属第一医院病理科; Department of Pathology, the First Affiliated Hospital of Soochow University, Suzhou 215006, China

**Keywords:** 原发性肺淋巴上皮癌, 亚型, 预后, Primary pulmonary lymphoepithelial carcinoma, Subtype, Prognosis

## Abstract

**背景与目的:**

原发性肺淋巴上皮癌（primary pulmonary lymphoepithelial carcinoma, PPLEC）是一种罕见的肺部恶性肿瘤，其发病率仅占所有肺恶性肿瘤的0.7%。目前，PPLEC被归类为鳞状细胞癌的特殊亚群。本研究旨在探讨PPLEC的两种亚型在临床病理学上的特征，以提升对该疾病的认识和诊断能力。

**方法:**

回顾性分析2019年2月至2023年6月苏州大学附属第一医院经病理学证实为PPLEC的14例患者的临床、病理学、影像学及预后资料，并对相关文献进行复习。

**结果:**

PPLEC总计14例，其中Regaud型5例，年龄33-73岁，男性2例，女性3例；Schmincke型9例，年龄36-79岁，男性4例，女性5例。计算机断层扫描（computed tomography, CT）均表现为软组织肿块影或结节影，Reagud型以周围型肿块为主，Schmincke型以中央型为主。病理学示肿瘤细胞呈合胞体样生长，伴随淋巴细胞浸润及间质纤维化，Regaud型边界清晰合并肉芽肿性炎性病变，Schmincke型肿瘤边界模糊。免疫组织化学示肿瘤细胞CK、CK5/6、P40、P63阳性，Regaud型Ki-67增殖指数低于Schmincke型；其中8例接受程序性细胞死亡配体1（programmed death-ligand 1, PD-L1）检测均呈阳性；EB病毒编码的小RNA（Epstein-Barr virus-encoded RNA, EBER）原位杂交检测均阳性；在所有病例中，6例接受手术治疗，8例实施综合治疗，截至随访结束14例患者均存活。

**结论:**

PPLEC是一种罕见的肺恶性肿瘤，与EB病毒（Epstein-Barr virus, EBV）感染相关。该疾病可分为Regaud型和Schmincke型，二者在影像学和病理学特征上具有独特差异。在治疗方面，早期以手术治疗为主，中晚期则采取综合治疗，整体预后较好。对于PD-L1高表达的中晚期患者，免疫治疗是一种较为有效的治疗选择。

## Abstract (en)

**Background and objective:**

Primary pulmonary lymphoepithelial carcinoma (PPLEC) is a rare form of lung malignancy, accounting for only 0.7% of all lung cancers. It is currently classified as a distinct subtype within squamous cell carcinomas. This study aims to explore the clinicopathological characteristics of PPLEC and its subtypes, with the objective of enhancing understanding and improving diagnostic accuracy for this disease.

**Methods:**

A retrospective analysis was conducted on the clinical, pathological, imaging, and prognostic data of 14 patients diagnosed with PPLEC at the First Affiliated Hospital of Soochow University between February 2019 and June 2023.

**Results:**

A total of 14 cases of PPLEC were identified, including 5 cases of the Regaud type, with ages ranging from 33 to 73 years, comprising 2 males and 3 females; and 9 cases of the Schmincke type, with ages ranging from 36 to 79 years, including 4 males and 5 females. Computed tomography (CT) scans consistently demonstrated soft tissue masses or nodular shadows. Reagud type mainly showed peripheral masses and Schmincke type mainly showed central masses. Pathological examination revealed tumor cells exhibiting syncytial-like growth, accompanied by lymphocytic infiltration and stromal fibrosis, with the Regaud type showing well-defined borders combined with granulomatous inflammation, while the Schmincke type exhibited indistinct tumor margins. Immunohistochemistry showed that CK, CK5/6, P40 and P63 were positive, and the Ki-67 index of Regaud type was lower than that of Schmincke type; notably, all 8 cases tested for programmed death-ligand 1 (PD-L1) were positive. Epstein-Barr virus-encoded RNA (EBER) in situ hybridization was positive in all instances. Among these cases, 6 underwent surgical treatment, and 8 received comprehensive therapy; by the end of the follow-up period, all 14 patients remained alive.

**Conclusion:**

PPLEC is a rare form of malignant lung tumor associated with Epstein-Barr virus (EBV) infection. The Regaud and Schmincke subtypes display distinct imaging and pathological characteristics. In the early stages of the disease, surgical intervention is the primary treatment method; however, for advanced stages, a multimodal treatment approach is utilized, resulting in a relatively favorable prognosis. Immunotherapy represents a promising and effective treatment modality for patients with middle to advanced stage disease exhibiting high PD-L1 expression levels.

原发性肺淋巴上皮癌（primary pulmonary lymphoepithelial carcinoma, PPLEC）是一种罕见的肺部恶性肿瘤，其发病率仅占所有肺恶性肿瘤的0.7%^[1]^，好发于我国南方地区及东南亚国家，与EB病毒（Epstein-Barr virus, EBV）感染密切相关^[2]^，通常与吸烟史及性别无关。根据2021年世界卫生组织第5版胸部肿瘤分类，PPLEC被归类为鳞状细胞癌的特殊亚群^[3]^，在形态学上，PPLEC与未分化鼻咽癌相似，有文献^[4,5]^报道，PPLEC可根据组织形态学特征分为Regaud型和Schmincke型。目前PPLEC缺乏标准化治疗方案，依赖于肺鳞癌的相关治疗经验，多采用手术治疗辅以放化疗手段^[6]^。由于PPLEC发病率低，缺乏大样本的数据，针对两种亚型在临床病理、治疗预后等方面特征的研究十分少见，本研究回顾性分析2019年2月至2023年6月经病理确诊的14例PPLEC，旨在探讨两种亚型的临床病理特征、影像学特征、免疫表型及治疗预后，以提高对PPLEC两种亚型的认知和诊断能力。

## 1 资料与方法

### 1.1 临床资料

收集2019年2月至2023年6月苏州大学附属第一医院经病理学证实为PPLEC的患者14例。所有患者均接受鼻咽内镜检查，排除鼻咽部转移性淋巴上皮瘤样癌。肿瘤分期以国际肺癌研究协会颁布的第8版分期为标准^[7]^。患者随访信息通过住院病历资料、门诊资料及电话随访获得，截止日期为2024年8月。本研究获我院伦理委员会批准[（2024）伦研批第370号]。

### 1.2 治疗方法

14例PPLEC患者中，6例患者接受肺癌根治手术治疗，8例患者接受综合治疗。

### 1.3 病理学检查

#### 1.3.1 常规苏木素-伊红（hematoxylin-eosin, HE）染色

标本离体后经10%中性福尔马林液固定，全自动组织处理仪（DAKEWE）处理，常规石蜡包埋，4 μm厚度连续切片，HE染色，中性树胶封片。

#### 1.3.2 免疫组织化学染色

采用EnVision两步法，CK、CK5/6、CK7、CgA、CD56、Syn、S-100、程序性细胞死亡配体1（programmed death-ligand 1, PD-L1）一抗购自DAKO公司，P40、P63、TTF-1、Napsin A、Ki-67一抗购自上海基因生物公司。其中PD-L1选用克隆号为22C3（DAKO Ominis平台），肿瘤细胞阳性比例评分（tumor cell proportion score, TPS）≥1%定义为PD-L1阳性。

#### 1.3.3 EB病毒编码的小RNA（Epstein-Barr virus-encoded RNA, EBER）原位杂交检测

探针及试剂检测盒购自北京中杉金桥生物技术有限公司。

### 1.4 结果判读

由两位病理医生单独阅片，差异较大则由第三位病理医生复片。根据镜下PPLEC癌巢与周围淋巴细胞和炎细胞浸润的特点将其分为Regaud型和Schmincke型，前者表现为癌巢结构明显，癌巢周围有大量淋巴细胞、炎细胞包绕；后者表现为癌巢结构不明显，癌细胞被淋巴细胞、炎细胞分开，散布在炎细胞之间^[4,5,8]^。

### 1.5 统计学方法

应用SPSS 20.0进行数据分析，利用双侧t检验分析Regaud型和Schmincke型肿块直径和Ki-67增殖指数的差异，利用Fisher精确检验分析Regaud型和Schmincke型在影像学分类上的差异，P<0.05为差异有统计学意义。

## 2 结果

### 2.1 临床特征及影像学表现

14例PPLEC中，Regaud型占5例，其中男性2例，女性3例，年龄范围33-73岁，平均年龄59岁，病灶部位：左肺3例，右肺2例，肿块直径范围1.3-5.3 cm，平均直径2.8 cm，肿块边界清晰，且边缘呈分叶状或毛刺状，其中4例表现为周围型肿块，1例表现为中央型肿块。Schmincke型有9例，其中男性4例，女性5例，年龄范围36-79岁，平均年龄61.1岁，病灶部位：右肺5例，左肺4例，肿块直径范围1.6-6.8 cm，平均肿瘤直径3.8 cm，其中2例边界模糊并伴有周围阻塞性肺炎。Regaud型以周围型肿块为主，Schmincke型以中央型肿块为主，差异有统计学意义（P=0.036），两种亚型肿块直径无明显差异（Regaud: 2.820±0.715 cm vs Schmincke: 3.778±0.508 cm, P=0.290）。此外，1例Schmincke型患者影像出现血管包埋征象，3例Schmincke型患者影像显示肿块累及胸膜（表1、表2及图1）。

**表1:** 14例PPLEC患者临床特征

Case	Gender	Age (yr)	Native place	Symptom	Smoking history	Stage	Subtype	Treatment	Follow-up results
1	F	69	Jiangsu	Cough and fever	N	IVA	Regaud	S	Survival
2	M	73	Jiangsu	N	Y	IIIA	Regaud	S+CT	Survival
3	F	33	Guangxi	N	N	IA	Regaud	S	Survival
4	F	56	Jiangsu	Thoracodynia	N	IA	Regaud	S	Survival
5	M	64	Jiangsu	Fever, cough and phlegm	N	IIB	Regaud	S+CT	Survival
6	M	71	Jiangsu	N	N	IIB	Schmincke	S+CT	Survival
7	F	61	Sichuan	Fecal deformity	N	IIIB	Schmincke	CT+I	Survival
8	M	49	Jiangsu	N	Y	IA	Schmincke	S+CT	Survival
9	F	52	Jiangsu	Abdominal pain with nausea and vomiting	N	IVA	Schmincke	S+CT+I	Survival
10	M	36	Anhui	Cough and sputum	N	IIIB	Schmincke	NCT+S+I	Recurrence and metastasis
11	F	79	Jiangsu	N	N	IA	Schmincke	S	Survival
12	M	72	Jiangsu	N	N	IA	Schmincke	S	Survival
13	F	71	Jiangsu	N	N	IB	Schmincke	S	Survival
14	F	59	Jiangsu	N	N	IVA	Schmincke	S+CT	Survival

F: female; M: male; Y: yes; N: No; S: surgery; NCT: neoadjuvant chemotherapy; CT: chemotherapy; I: immunotherapy; PPLEC: primary pulmonary lymphoepithelial carcinoma.

**表2:** 14例PPLEC患者影像学表现

Case	Tumor location	Type	Tumor size (mm)	Leaflet	Burr	Boundary	Calcification	Obstructive pneumonia	Type
1	RUL	P	24×15	Y	N	D	N	N	Regaud
2	LUL	C	34×24	N	Y	D	N	N	Regaud
3	LUL	P	17×13	Y	N	D	N	N	Regaud
4	LLL	P	13×13	Y	Y	D	N	N	Regaud
5	RML	P	53×48	N	Y	D	N	N	Regaud
6	RLL	C	46×45	Y	Y	D	N	N	Schmincke
7	LUL	C	43×26	Y	Y	B	Y	Y	Schmincke
8	RLL	C	29×22	Y	Y	D	N	N	Schmincke
9	LLL	P	43×35	Y	Y	D	N	N	Schmincke
10	LLL	C	68×42	Y	N	B	N	Y	Schmincke
11	LLL	C	26×23	Y	N	D	N	N	Schmincke
12	RML	P	11×16	Y	Y	D	N	N	Schmincke
13	RLL	C	42×25	Y	N	D	N	N	Schmincke
14	RML	C	27×19	Y	N	D	N	N	Schmincke

RUL: right upper lobe of lung; RML: right middle lobe of lung; RLL: right lower lobe of lung; LUL: left upper lobe of lung; LLL: left lower lobe of lung; P: peripheral type; C: central type; D: distinct; B: blur.

**图1:**
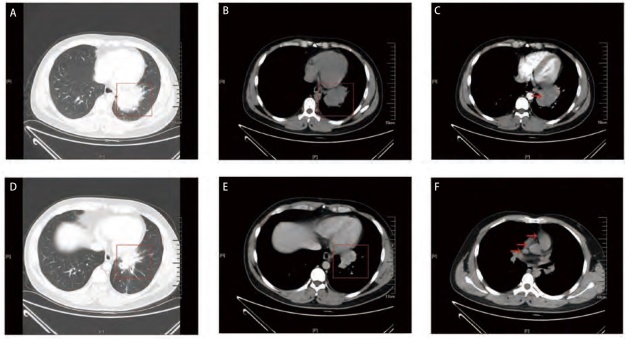
病例10影像学表现。A-C：病例10，男性，36岁，CT显示左下肺靠近肺门肿块，分叶状，68 mm×42 mm，边界模糊，增强CT显示呈均匀低密度实变的肺部肿块内可见强化肺血管成分；D、E：病例10经过2个周期化疗后复查，左下肺叶靠近肺门肿块，49 mm×30 mm，分叶状，边界欠清，肿块较之前缩小；F：纵隔窗红色箭头所指显示纵隔淋巴结肿大。

### 2.2 病理学特征

Regaud型肉眼观察切面呈灰白或灰红色，显微镜下观察发现，该类型细胞的边界清楚，周围包绕着淋巴细胞和浆细胞，且可以见到肉芽肿性炎性病变。在部分病例中，能够观察到虫卵及钙化灶（图2），其中1例Regaud型患者的淋巴结出现癌转移；相较之下，Schmincke型的切面呈灰白、灰褐或灰黑色，显微镜下，细胞呈现弥漫生长，癌细胞与炎细胞交杂在一起，且被淋巴细胞和浆细胞所隔离，细胞的边界模糊（图2）。在Schmincke型病例中，有2例淋巴结出现癌转移并且1例Schmincke型癌症已经侵犯到胸壁组织。

**图2:**
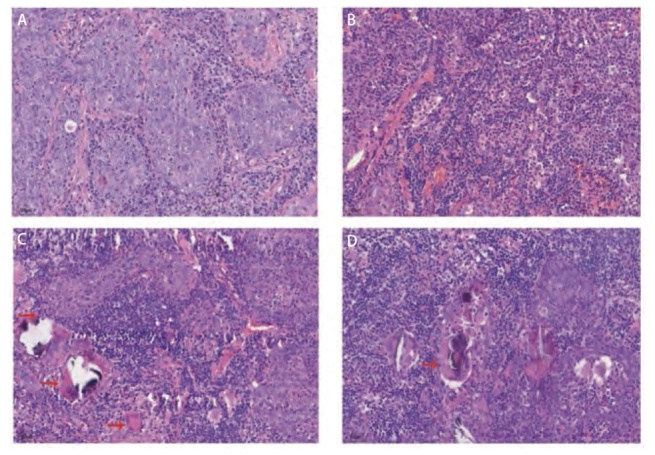
PPLEC病理学表现（HE，×400）。A：Regaud型细胞巢状生长，周围包绕大量炎细胞，边界清晰，间质可见纤维组织；B：Schmincke型细胞弥漫生长，与炎细胞边界不清；C：红色箭头所指为肉芽肿性炎性病变肉芽肿性炎性病变及钙化灶；D：红色箭头所指为吞噬虫卵的巨噬细胞。

### 2.3 免疫表型及原位杂交

Regaud型免疫组化结果（图3）显示，其鳞状细胞表型标志物CK、CK5/6、P40和P63均呈阳性，而腺上皮来源的标志物Napsin A、TTF-1则不表达。此外，神经内分泌标志物CgA、Syn及CD56均呈阴性。值得注意的是，在1例患者中，肿瘤细胞周围CD20及CD3淋巴细胞呈散在阳性表达（图4），CD3阳性细胞数量多于CD20阳性细胞数量。Ki-67平均增殖指数为53.00%±10.68%。仅有1例Regaud型患者接受了PD-L1检测，结果显示TPS为10%；Schmincke型肿瘤细胞表现出CK、CK5/6、P40和P63等鳞状细胞表型标志物的阳性反应，Napsin A、TTF-1等腺上皮来源标志物基本不表达，仅1例Schmincke型TTF-1呈部分弱阳性，在神经内分泌标志物方面，CgA、Syn及CD56均为阴性，2例患者肿瘤细胞周围CD20及CD3淋巴细胞也呈散在阳性表达（图4），与Regaud型表达类似，CD3阳性细胞数量多于CD20阳性细胞数量。Ki-67平均增殖指数为66.67%±2.89%，Regaud型增殖指数低于Schmincke型（P=0.016），7例Schmincke型患者接受PD-L1检测，其中5例患者PD-L1的TPS>50%，1例患者的TPS=50%。所有样本的EBER原位杂交检测结果均为阳性（表3）。

**图3:**
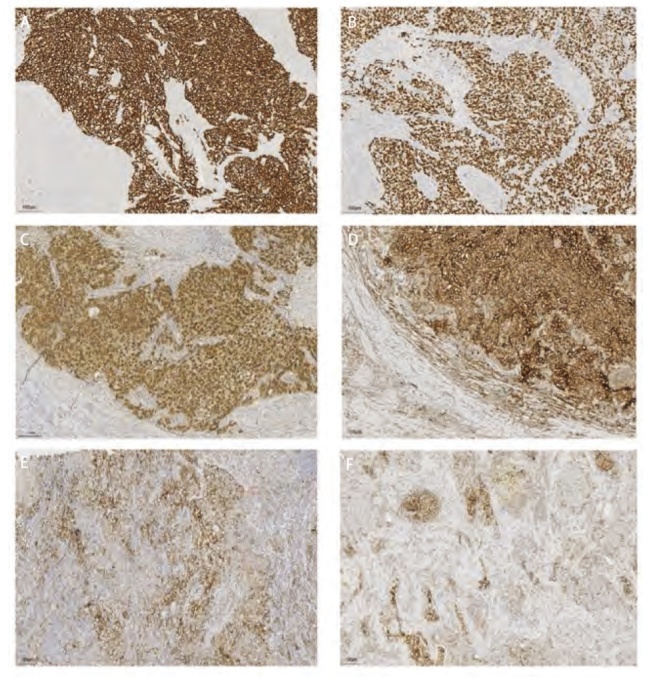
部分免疫组化、EBER及PD-L1结果（IHC，×200）。A：CK5/6阳性；B：P40阳性；C：EBER阳性；D：PD-L1≥50%，约为90%；E：PD-L1约为50%；F：PD-L1约为10%。

**图4:**
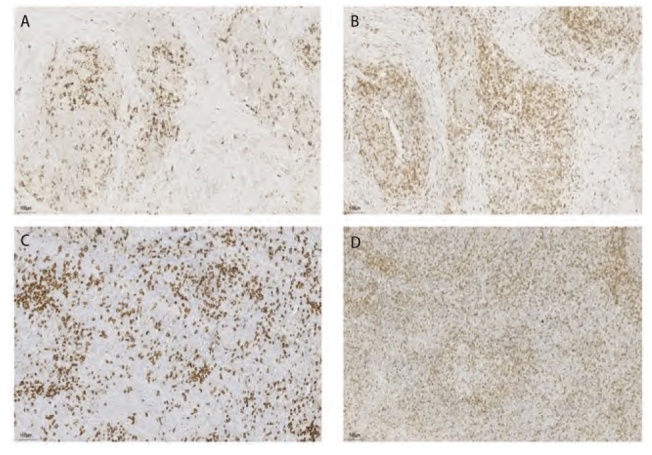
CD20和CD3免疫组化结果（IHC, ×200）。A：Regaud型CD20淋巴散在阳性；B：Reagud型CD3淋巴细胞散在阳性；C：Schmincke型CD20散在阳性；D：Schmincke型CD3散在阳性。

**表3:** 14例PPLEC免疫组化、PD-L1及EBER检测结果

Case	CK	CK5/6	P40	P63	Napsin A	CgA	Syn	CD56	TTF-1	Ki-67	PD-L1	EBER
1	NA	+	+	+	NA	NA	-	-	-	50%+	NA	+
2	+	+	+	+	-	-	-	-	-	90%+	10%	+
3	+	+	+	NA	-	-	-	-	-	60%+	NA	+
4	+	+	+	+	-	NA	NA	NA	-	35%+	NA	+
5	NA	+	+	NA	NA	NA	NA	NA	NA	30%+	NA	+
6	+	+	+	+	-	-	-	-	-	70%+	NA	+
7	NA	+	+	+	-	NA	-	NA	±	70%+	1%	+
8	+	+	+	+	-	-	-	NA	-	60%+	60%	+
9	NA	+	+	+	-	NA	NA	NA	-	60%+	NA	+
10	NA	+	+	+	NA	NA	NA	NA	-	80%+	90%	+
11	+	+	+	+	-	NA	NA	NA	-	60%+	90%	+
12	+	+	+	-	-	NA	NA	NA	-	80%+	90%	+
13	NA	+	+	NA	NA	NA	NA	NA	-	60%+	>50%	+
14	+	+	+	NA	-	-	-	-	-	60%+	50%	+

NA: not available; +: positive; -: negative.

### 2.4 治疗及预后

6例患者接受肺癌根治手术治疗，8例患者则采用综合治疗方案，其中化疗主要使用紫杉醇或吉西他滨联合铂类药物，而免疫治疗则选用信迪力单抗或替雷利珠单抗。在治疗期间，3例Schmincke型患者出现骨髓抑制，表现出不同的临床症状，包括皮肤红疹、肝功能不全、肾功能不全等；还有1例Schmincke型患者则出现肝脏及腹膜后多发转移。通过门诊复查、住院病历查询以及电话随访，截至2024年8月，所有患者均存活。

## 3 讨论

PPLEC是一种罕见的特殊肺鳞状细胞癌亚型^[3,9,10]^，其发病率不足总体肺癌发病率的1%^[1,6,11]^。研究^[1,10,12]^指出，PPLEC分布存在显著地域性倾向，以东亚尤其是中国南部地区为高发区域，该疾病在特定人群的聚集现象尤为明显，非吸烟者及年轻亚洲女性是主要患病群体^[5,6]^。PPLEC的发病年龄范围集中在51-55岁，低于其他类型肺癌^[5,10]^。在本研究中，无吸烟史者的患病率更高，符合以往研究结果^[13]^，但本研究中男女发病比率相近，可能与研究数据较少有关。在本研究中PPLEC两种亚型的平均发病年龄均高于已报道范围，这可能归因于苏南地区优越的经济条件、先进的医疗环境以及民众健康意识的普遍提升。此外，大部分患者在就诊时无明显症状，少数患者出现发热、咳嗽咳痰、胸痛和胃肠道不适等，表明PPLEC患者早期临床表现不典型，需依靠其他检查手段辅助确诊。

近年来，CT已经成为肺部影像学检查的首选方式，既往研究^[14]^显示PPLEC多表现为体积较大、边界清晰、伴有分叶的单一肿块，并可伴有血管或支气管包埋征象，偶可见肿瘤钙化灶。本研究中，影像学显示Regaud型主要为周围型单发肿块，平均直径为2.8 cm，边界清晰；而Schmincke型则主要呈现中央型单发肿块，其肿块平均直径为3.8 cm，可伴有周围阻塞性肺炎及钙化现象，且肿块可侵及胸膜及血管。上述影像学表现与既往研究结果^[14]^类似，提示我们影像学检查可为诊断PPLEC及区分两种亚型提供一定帮助，但仍需更多临床报道以验证这一发现。

病理学是诊断PPLEC的金标准。肿瘤大体观察显示，两种亚型均表现为单一较大肿块，质实而较硬，且肿块紧邻或接近胸膜。显微镜下，瘤细胞呈合体状聚集，核大、空泡状，核仁嗜酸，肿瘤实质和间质均伴有大量淋巴细胞及浆细胞浸润，以成熟淋巴细胞为主，间质可见大量纤维组织及胶原。Regaud型肿瘤细胞呈巢状生长，边界清晰，周边有淋巴细胞及浆细胞包裹，常见肉芽肿性炎性病变、虫卵及钙化灶。而Schmincke型肿瘤细胞则呈弥漫片状生长，瘤细胞与淋巴细胞混合存在，边界不清。尽管病理学在诊断及区分两种亚型中发挥重要作用，但目前对此方向的研究较少，尚缺乏明确诊断标准。因此，我们的研究可为PPLEC的亚型分类提供新的依据。

免疫组化结果显示，两种亚型的肿瘤细胞均表达鳞状上皮标记。既往研究^[15,16]^表明，PPLEC中浸润的淋巴细胞主要为CD8^+^和TIA^+^的T细胞，Kobayashi等^[15]^通过免疫组化双染证实这一类T细胞的浸润与HLA^-^DR^+^PPLEC细胞密切相关；Chang等^[17]^则认为这类T细胞数量的增多可能是特异于EBV诱导的细胞基因编码的肿瘤细胞抗原。而本组数据中，在1例Regaud型和2例Schmincke型患者中，CD20和CD3均显示出散在的淋巴细胞阳性表达。其中，CD3阳性细胞数量明显高于CD20阳性细胞数量，这提示存在以T淋巴细胞为主的混杂性增生。然而，由于标本量有限及部分标本不可得，我们无法进一步分析这两种亚型肿瘤微环境中的免疫细胞差异。因此，我们计划在后续研究中扩大样本量，以深入探讨浸润免疫细胞之间的差异并进一步分析相关机制。两种亚型的Ki-67增殖指数介于30%-90%之间，其中Schmincke型的平均增殖指数明显高于Regaud型（P=0.016）。研究^[18]^表明，Ki-67增殖指数与肿瘤的侵袭性和远处转移风险密切相关。Regaud型的癌巢结构清晰，癌巢之间有大量淋巴细胞及浆细胞浸润，影像学表现也较为明确，相比之下，Schmincke型的癌巢结构不明显，癌细胞被淋巴细胞及浆细胞分开，散布于炎细胞之间，组织学类似于霍奇金淋巴瘤，这种分散的结构可能使得Schmincke型更容易形成空洞，从而增加肿瘤的侵袭性和转移风险^[19]^。因此较高的Ki-67增殖指数及特殊的病理形态使Schmincke型更易于发生转移、侵袭等变化，但关于Reagud型和Schmincke型在肿瘤转移和侵袭性方面的具体研究仍不足，亟需进一步探讨。此外，本研究中所有患者的EBER检测均为阳性，表明EBER检测可作为PPLEC的重要鉴别诊断指标。

迄今尚无针对PPLEC的标准治疗方案，临床通常采用综合治疗策略^[20]^。近年来，免疫治疗被视作极具前景的治疗方式，可延长转移性或复发患者的生存期，并显著提高其生活质量^[21]^。PD-L1作为非小细胞肺癌细胞表面抑制性分子，与程序性细胞死亡蛋白1（programmed cell death-1, PD-1）受体结合，抑制T细胞增殖和活化^[22]^，因此，阻断PD-1/PD-L1通路为免疫治疗开辟了新途径。相关研究^[1]^表明，PPLEC中PD-L1表达显著高于其他类型的肺癌，超过半数患者的PD-L1的TPS>50%，这些PD-L1阳性患者的总体生存期与无进展生存期均优于阴性患者，同时对PD-L1免疫抑制剂表现出良好的疗效。因此，PD-L1表达水平较高并有较多淋巴细胞浸润的PPLEC患者将可能是免疫治疗的潜在获益人群^[1,23⇓-25]^。本研究中，Regaud型多采用手术及化疗手段治疗，预后较好。早期Schmincke型患者采用手术和化疗治疗，而PD-L1高表达的中晚期Schmincke型患者则采用手术、化疗和免疫治疗。例如病例10为PD-L1高表达的中晚期Schmincke型PPLEC，术前患者表现为左肺下叶近肺门处巨大肿瘤（68 mm×42 mm），纵隔多发肿大淋巴结，患者先接受吉西他滨加卡铂化疗方案，并联合使用信迪力单抗进行2个周期的免疫治疗，影像学检查显示左肺下叶肿块缩小（49 mm×30 mm）后，患者接受了肺癌根治手术，术后继续进行相同的化疗和免疫治疗共9个周期。在此期间，患者胸腔积液略有吸收，然而在治疗3个月后，双肺多发小结节增加，1年后则出现肝脏及腹膜后多发转移现象，患者随即住院进行治疗。这表明，尽管免疫治疗在一定程度上显示出缩小肿块、吸收胸腔积液等积极变化，但长期治疗可能会导致耐药并引发转移等不良后果；同时也提示我们患者术前肿瘤T分期以及淋巴结转移情况可能会对免疫治疗的效果有所影响。研究^[10]^表明，约60%的晚期PPLEC患者通过免疫治疗获得病情稳定，但也有约25%的PPLEC患者在治疗20个月后对PD-L1抑制剂出现耐药^[5,24]^。有研究^[24]^指出，免疫治疗的有效性不仅取决于PD-L1在肿瘤细胞中的表达，还受肿瘤微环境中免疫效应细胞数量的影响。但由于本研究的样本量有限，我们的数据显示，PPLEC中T细胞和B细胞混杂性增生，CD3阳性细胞数量多于CD20阳性细胞，尚需进一步扩大样本量以分析两种亚型间肿瘤微环境的差异。此外，本研究中接受免疫治疗的3例患者病理分型均为Schmincke型，病理学亚型是否影响免疫治疗的效果亟需进一步研究。

Schmincke型PPLEC具有弥漫分布特征，癌细胞散布在炎细胞之间，相较于Regaud型，更容易发生淋巴结及其他器官转移和侵袭，因此，可能需要更积极的治疗策略以改善预后。ALK、KRAS、EGFR等常见肺癌基因突变在PPLEC中较为罕见^[20,24]^。例如，Zhang等^[12]^在11例PPLEC病例中仅发现3例患者携带基因突变：1例为CYP2D6联合UGT1A1突变，1例为PAK3突变，1例为TP53突变。这表明晚期PPLEC患者从靶向治疗中获益甚微，因此免疫治疗仍是中晚期PPLEC，尤其是PD-L1高表达Schmincke型患者的优选。此外，其他资料也支持这一观点。例如，ALK基因重排在PPLEC中非常罕见，大多数研究^[26]^中未检测到ALK基因重排，同时，KRAS突变在PPLEC中也未被发现，这些基因突变的罕见性进一步说明了PPLEC患者对传统靶向治疗的低反应性。尽管如此，PD-L1表达水平较高（超过50%）的PPLEC患者可能从免疫治疗中受益，因为PD-L1高表达与免疫治疗的有效性相关^[10]^。综上所述，由于ALK、KRAS、EGFR等常见肺癌基因突变在PPLEC中的罕见性，晚期PPLEC患者从靶向治疗中获益有限，而免疫治疗尤其是针对PD-L1高表达患者的治疗策略显得更为重要。

这项研究存在一些不足，其中最明显的问题是样本量过小。由于PPLEC的低发病率，2019年2月至2023年6月本院仅14例患者被诊断为PPLEC，而每年新诊断的肺癌病例超过5000例。此外，免疫疗法作为一种相对较新的治疗手段，在这14例 PPLEC患者中，仅3例接受了不同治疗方案的免疫检查点抑制剂。不同治疗方案之间的异质性还有待进一步探讨。值得注意的是，本研究中的所有患者分子生物学特征均较为罕见，这对我们深入探讨PPLEC的分子机制及靶向治疗的研究造成了困难。因此，我们亟需扩大样本量，以便开展更为系统的研究。未来，我们计划进行一项观察期足够且涵盖多个中心的研究，以提供更具说服力的证据。

综上所述，PPLEC作为一种罕见的恶性肿瘤，多见于中国南方，以非吸烟和60岁左右的患者为主要发病对象。影像学和病理学在鉴别诊断PPLEC并区分两种亚型方面具有重要作用。早期PPLEC主要采用手术治疗，而中晚期尤其是PD-L1高表达的Schmincke型患者则首选手术并辅以化疗及免疫治疗的综合治疗方案，预后较好。

## References

[1] Archwamety A, Ruangchira‐urai R, Akewanlop C, et al. Primary pulmonary lymphoepithelioma‐like carcinoma treated with immunotherapy: A case report and literature review. Thorac Cancer, 2022, 13(17): 2539-2541. doi: 10.1111/1759-7714.14580 35830974 PMC9436678

[2] Bégin LR, Eskandari J, Joncas J, et al. Epstein-Barr virus related lymphoepithelioma-like carcinoma of lung. J Surg Oncol, 1987, 36(4): 280-283. doi: 10.1002/jso.2930360413 2826922

[3] Nicholson AG, Tsao MS, Beasley MB, et al. The 2021 WHO classification of lung tumors: impact of advances since 2015. J Thorac Oncol, 2022, 17(3): 362-387. doi: 10.1016/j.jtho.2021.11.003 34808341

[4] Nogal P, Staśkiewicz M, Jackowska J, et al. Lymphepithelial carcinoma - a rare tumor of the larynx. Case Report. Front Surg, 2022, 9: 851481. doi: 10.3389/fsurg.2022.851481 PMC9650521 36386509

[5] Fan Y, Li C, Qin J, et al. Primary pulmonary lymphoepithelioma-like carcinoma. Med Oncol, 2020, 37(3): 20. doi: 10.1007/s12032-020-1344-3 32146584

[6] Sheng H, He X, Chen Z, et al. Development of a haematological indices-based nomogram for prognostic prediction and immunotherapy response assessment in primary pulmonary lymphoepithelioma-like carcinoma patients. Transl Lung Cancer Res, 2024, 13(3): 453-464. doi: 10.21037/tlcr-23-813 38601436 PMC11002515

[7] Detterbeck FC, Boffa DJ, Kim AW, et al. The eighth edition lung cancer stage classification. Chest, 2017, 151(1): 193-203. doi: 10.1016/j.chest.2016.10.010 27780786

[8] Saw D, Ho JH, Fong M, et al. Prognosis and histology in stage I nasopharyngeal carcinoma (NPC). Int J Radiat Oncol Biol Phys, 1985, 11(5): 893-898. doi: 10.1016/0360-3016(85)90110-5 3988561

[9] Ho JC, Wong MP, Lam WK. Lymphoepithelioma‐like carcinoma of the lung. Respirology, 2006, 11(5): 539-545. doi: 10.1111/j.1440-1843.2006.00910.x 16916325

[10] Low YH, Loh CJL, Peh DYY, et al. Pathogenesis and therapeutic implications of EBV-associated epithelial cancers. Front Oncol, 2023, 13: 1202117. doi: 10.3389/fonc.2023.1202117 PMC10600384 37901329

[11] Bao H, Ma LZ, Zhao C, et al. Anti-angiogenic therapy for advanced primary pulmonary lymphoepithelioma-like carcinoma: a retrospective multicenter study. J Cancer Res Clin Oncol, 2022, 149(3): 1185-1193. doi: 10.1007/s00432-022-03935-0 35377040 PMC9984323

[12] Zhang Q, Dai Y, Jin L, et al. Clinicopathological characteristics and cancer-specific prognosis of primary pulmonary lymphoepithelioma-like carcinoma: a population study of the US SEER database and a Chinese hospital. Front Oncol, 2023, 13: 1103169. doi: 10.3389/fonc.2023.1103169 PMC10235615 37274245

[13] He J, Shen J, Pan H, et al. Pulmonary lymphoepithelioma-like carcinoma: a Surveillance, Epidemiology, and End Results database analysis. J Thorac Dis, 2015, 7(12): 2330-2338. doi: 10.3978/j.issn.2072-1439.2015.12.62 26793355 PMC4703678

[14] Pan D, You B, Lin HS, et al. CT features of primary pulmonary lymphoepithelial carcinoma. Shiyong Yixue Yingxiang Zazhi, 2023, 24(5): 325-328.

[15] Kobayashi M, Ito M, Sano K, et al. Pulmonary lymphoepithelioma-like carcinoma: predominant infiltration of tumor-associated cytotoxic T lymphocytes might represent the enhanced tumor immunity. Intern Med, 2004, 43(4): 323-326. doi: 10.2169/internalmedicine.43.323 15168777

[16] Maniglio S, Cazzato G, Caporusso C, et al. Poorly differentiated cutaneous squamous cell carcinoma (cSCC) or lymphoepithelioma-like carcinoma of the skin (LELCS) with squamous pearls: a case presentation with emphasis on histomorphological features and classification debates. Life (Basel), 2023, 13(12): 2265. doi: 10.3390/life13122265 PMC10744854 38137866

[17] Chang YL, Wu CT, Shih JY, et al. New aspects in clinicopathologic and oncogene studies of 23 pulmonary lymphoepithelioma-like carcinomas. Am J Surg Pathol, 2002, 26(6): 715-723. doi: 10.1097/00000478-200206000-00004 12023575

[18] Andrés-Sánchez N, Fisher D, Krasinska L. Physiological functions and roles in cancer of the proliferation marker Ki-67. J Cell Sci, 2022, 135(11): jcs258932. doi: 10.1242/jcs.258932 35674256

[19] Chen YS, Miao Y, Li HR, et al. A case of primary lung lymphoepitheliomatoid carcinoma. Zhonghua Jiehe He Huxi Zazhi, 2013, 36(1): 61-63.

[20] Hsieh JCH, Tang L, Chen N, et al. The clinicopathological features and prognosis of primary pulmonary lymphoepithelioma-like carcinoma: A systematic review and meta-analysis. PLoS One, 2020, 15(10): e0240729. doi: 10.1371/journal.pone.0240729 PMC7567369 33064745

[21] Yu XY, Zhang XW, Wang F, et al. Correlation and prognostic significance of PD-L1 and P 53 expression in resected primary pulmonary lymphoepithelioma-like carcinoma. J Thorac Dis, 2018, 10(3): 1891-1902. doi: 10.21037/jtd.2018.03.14 29707344 PMC5906333

[22] Wu Z, Xian X, Wang K, et al. Immune checkpoint blockade therapy may be a feasible option for primary pulmonary lymphoepithelioma-like carcinoma. Front Oncol, 2021, 11: 626566. doi: 10.3389/fonc.2021.626566 33981599 PMC8110193

[23] Chang YL, Yang CY, Lin MW, et al. PD-L1 is highly expressed in lung lymphoepithelioma-like carcinoma: A potential rationale for immunotherapy. Lung Cancer, 2015, 88(3): 254-259. doi: 10.1016/j.lungcan.2015.03.017 25862146

[24] Fan Y, Shan Q, Gong J, et al. Molecular and clinical characteristics of primary pulmonary lymphoepithelioma-like carcinoma. Front Mol Biosci, 2021, 8: 736940. doi: 10.3389/fmolb.2021.736940 34760925 PMC8573970

[25] Yin K. Prognostic significance of immunomolecular markers in primary lung lymphoepitheliomatoid carcinoma and prognostic significance of PD1/PD-L1 inhibitors. Guangdong: Southern Medical University, 2019.

[26] Hu Y, Ren S, Liu Y, et al. Pulmonary lymphoepithelioma-like carcinoma: A mini-review. Onco Targets Ther, 2020, 13: 3921-3929. doi: 10.2147/ott.S241337 32494151 PMC7227818

